# Ultrasound measurements of uterine height, horns diameter and presence of intraluminal fluid to investigate uterine involution in lactating sows housed in farrowing crates

**DOI:** 10.1590/1984-3143-AR2021-0066

**Published:** 2022-09-12

**Authors:** Pierre Thilmant, Dominiek Maes, Jean-François Beckers, Evelyne Moyse, Frédéric Farnir, Johann Detilleux, Martine Laitat

**Affiliations:** 1 Centre d'Insémination Artificielle Porcin, Argenteau, Liège, Belgium; 2 Vakgroep Voortplanting, Verloskunde en Bedrijfsdiergeneeskunde, Faculteit Diergeneeskunde, Universiteit Gent, Merelbeke, Belgium; 3 Département des Sciences Fonctionnelles, Faculté de Médecine Vétérinaire, Université de Liège, Liège, Belgium; 4 Département de Gestion Vétérinaire des Ressources Animales, Faculté de Médecine Vétérinaire, Université de Liège, Liège, Belgium; 5 Département Clinique des Animaux de Production, Faculté de Médecine Vétérinaire, Université de Liège, Liège, Belgium

**Keywords:** lactating sow, reproduction, swine, ultrasonography, uterine involution

## Abstract

The uterine involution of sows housed in farrowing crates was investigated during lactation using B-mode trans-abdominal ultrasonography. The objectives were to describe uterine involution, detect any delay or uterine disorders and assess possible associations between involution and subsequent reproductive performance. Three parameters were measured: uterine height (H), horns diameter (D) and the percentage of sows with intraluminal fluid (F). During lactation (3-4 weeks), H decreased from 11.0±1.6 the first week to 5.9±1.5 cm the last week (p<0.001), and D from 2.6±0.7 to 1.4±0.2 cm (p<0.001). Between days 1-7, H and D decreased significantly faster, i.e. respectively 0.38 cm (p<0.0001) and 0.20 cm (p<0.0001) per day than between days 22-28, i.e. respectively 0.02 cm (p=0.49) and 0.00 cm (p=0.75) per day. F decreased significantly (p<0.0001) from 78% at the beginning to 16% at the end of lactation. Between days 1-7, F decreased significantly (p<0.001) faster than during the last week of lactation (p=0.41). Between days 22-28, H of sows from parity ≥3 were significantly higher than those of sows from parity 1 and 2 (p=0.007). During that period, F was significantly higher in sows of higher parity. This effect of parity on F was significantly higher during the entire lactation period in sows of parity ≥6. Some sows were monitored after weaning. There was no significant relationship between the 3 parameters measured at the end of lactation and the subsequent performance. A small number of sows was suspected of endometritis (2%) and one case of fœtoplacental retention was detected. In conclusion, B-mode ultrasonography is a suitable tool to monitor uterine involution in lactating sows. When examination is conducted during the last week of lactation, it may help the farmer to verify whether uterine involution is complete, and to decide whether a sow should be either culled or maintained on farm.

## Introduction

Uterine involution is defined as the return, postpartum, of uterus to the non-gravid state following tissues degenerative and regenerative processes (Belstra et al., 2005). It prepares a suitable environment to establish the next pregnancy (Slama et al., 1999). Sows are characterized by a diffuse epitheliochorial placentation and a rapid uterine involution (2 to 3 weeks).

In the sow, the involution is nearly completed 18 days postpartum (Martinat-Botté et al., 1998) and uterine contractions are very strong postpartum (Kirkwood et al., 2012). They continue for at least two days. It is indeed normal to observe vulvar discharges (or lochia) during two days postpartum (Kirkwood et al., 2012). A normal sow’s lochia contents placental fluid, endometrial exudates and often absence of blood (Slama et al., 1999) and are visible during 3 to 4 days. The associated inflammatory reaction is acute and tissue reorganization is early (Slama et al., 1999).

Certain circumstances may delay the smooth progress of uterine involution. Egli et al. (2022) have shown that the body condition score, the gestation length and fever occurred during the first five days post farrowing have significant effects on uterine involution in sows in a free farrowing system. Assisted farrowing, increased farrowing duration, parity >5 and stillbirth are risk factors for prolonged post parturient vaginal discharge (Nguyen, 2020). Björkman and collaborators (Björkman, 2017; Björkman et al., 2018) have confirmed that prolonged parturition, impaired placenta expulsion, obstetrical intervention (i.e. manual palpation) and stillborn piglets increase the risk for intrauterine fluid accumulation and increased uterine size in the first week postpartum (2017). Postpartum metritis delays uterine involution whereas the use of exogenous oxytocin supports it (Björkman et al., 2018). Peltoniemi et al. (2016) suggest that connections exist among prolonged farrowing and yield of colostrum, retained placenta, development of postpartum dysgalactia syndrome, and impaired involution of the uterus and reduced subsequent fertility.

Transrectal ultrasonography and rectal palpation are frequently used to evaluate uterine involution for prediction of postpartal fertility in the cow (Aslan et al., 2002; Mee et al., 2009). Various techniques exist to evaluate uterine involution in sows, such as the titration of urinary excretion of collagen degradation markers (Belstra et al., 2005), uterine biopsy (Rummer and Elze, 1980) or ultrasonography (Martinat-Botté et al., 1998). Martinat-Botté et al. (1998) have measured the height occupied by the sow’s uterus in the ultrasound scan, or "uterine height", and showed that it decreases *i.e.* 7-10 cm the first week after parturition to 4-6 cm three weeks postpartum. Palmer et al. (1965) reported a similar evolution of the uterus weight during this period *i.e.* 3 Kg just postpartum and less than 500 grams 21 days postpartum. Delayed uterine involution 21-28 days postpartum could have a negative effect on sow’s productivity. Schnurrbusch et al. (2009) showed that the weight of the genital organs of breeding pigs culled and slaughtered because of infertility was higher than normal in 49% of the cases in gilts and in 71% of the cases in sows. Very frequently, they have noticed functional endometrial disorders that could be due to mycotoxins. Furthermore, endometritis with bacterial infections were registered in 23% and 37% of gilts and sows, respectively. The ultrasonography control could detect delayed uterine involution, abnormal fluid accumulation, metritis and/or pyometra (Björkman et al., 2018). It may help to decide whether sows should be treated or culled after weaning. The goal is to decrease the number of unproductive days of the herd.

The objectives were to describe uterine involution, to detect any delay or uterine disorders and to assess possible associations between involution and subsequent reproductive performance.

## Methods

The present research was approved by the Research Ethics Committee of University of Liège (approval number 21-2375).

### Animals and farm management

A total of 709 sows (parity 1-13), bred using artificial insemination, from 9 Walloon (Belgium) commercial pig herds were investigated. Farms were client farms of a veterinary practice, did not have significant reproductive problems in the sows, and were willing to collaborate. Sows were weaned between 21 to 28 days after parturition. Sixty-seven sows were excluded from the study because they had received an altrenogest treatment during lactation or because of insufficient or inconsistent data. Data available from 642 healthy females were used to describe uterine involution. In a way to study the relation between uterine involution and sows performance on the next breeding cycle, 428 weaned sows were further investigated. Eighteen females which were given a treatment based on both PMSG and HCG (PG600^®^, MSD A.H.) the day after weaning were excluded as well as 196 sows as they were not weaned and/or scanned 22-28 days postpartum. No information was available in the farm records whether animals had dystocia, received obstetric care and/or whether oxytocin was administered during parturition.

The females were essentially genetic type K+ (raised muscular hyper-prolific Belgian Landrace line; n = 369) or commercial maternal line (CML) 1 (n = 167). Sows from other genetic types were also present in some farms: CML 2 (n = 74), CML 3 (n = 28) and CML 4 (n = 4). In four farms, the genetic type K + was only present and in another one, all sows were CML 1. In other pig farms, several genetic types were present (Table 1).

**Table 1:** Breeding data for 642 sows from 9 farms examined by means of transcutaneous ultrasonography (Falco - Esaote Pie Medical - equipped with 5 MHz micro-convex probe) to describe uterine involution: number of sows per farm, number of scanned sows per farm, mean parity of scanned sows and systematic treatments. Genetic types were one raised type (Belgian Landrace K+) and 4 Commercial Maternal Lines (CML). The mean number of total born piglets (TBP, mean ± standard deviation) and the mean number of weaned piglets (mean ± SD) was measured for each sow during the study.

**Farms**	**Number of sows/farm (n)**	**Scanned sows (n)**	**Mean parity of scanned sows**	**Genetic type**	**Systematic Treatments**	**Mean TBP**	**Mean number of weaned piglets**
A	105	10	3.4 ± 2.0	CML 1 and 3	-	15.0 ± 2.3	11.5 ± 2.6
B	120	35	4.0 ± 2.5	Belgian Landrace K+	Dinolytic^®^a	11.8 ± 2.8	9.6 ± 2.2
C	120	11	4.9 ± 3.9	CML 1, 3 and 4	-	12.4 ± 4.1	8.5 ± 2.0
D	120	43	3.3 ± 2.3	CML 1 and 3	-	11.5 ± 3.5	10.1 ± 2.0
E	670	147	3.3 ± 2.0	CML 1, 2, 3 and 4	Dinolytic^®^b/PG600^®^*	12.3 ± 2.9	10.2 ± 1.8
F	200	62	4.4 ± 1.5	CML 1	-	11.5 ± 3.1	8.6 ± 2.0
G	340	196	4.1 ± 3.0	Belgian Landrace K+	Dinolytic^®^c	12.0 ± 3.0	9.7 ± 1.2
H	240	132	5.2 ± 3.1	Belgian Landrace K+	-	11.4 ± 3.3	9.2 ± 2.0	
I	70	6	5.2 ± 3.0	Belgian Landrace K+	-	9.2 ± 4.7	8.3 ± 2.7	

^a^Dinolytic^®^, all sows on day 113 of pregnancy; ^b^Dinolytic^®^, all sows on day 117 of pregnancy; ^c^Dinolytic^®^, all sows, 36 h postpartum; ^*^PG600^®^, all sows in September and October.

To test the effect of parity on uterine involution, 628 sows (with available data) were classified in three groups in order to have a greater number of individuals per category: group A, parity 1-2 (n=214); group B, parity 3-5 (n=242) and group C, parity ≥ 6 (n=172).

### Ultrasonographic examination of the uterus

Sows were examined three to four times by transcutaneous ultrasonography every 7 days during the lactation period (*i.e.* 2,325 ultrasound scans). Scanning was performed using the transportable ultrasound equipment FALCO (Esaote Pie Medical) adjusted to a 5 MHz micro-convex probe. The probe was positioned on the abdomen of the standing sow above the mammary chain and oriented to the bladder, as described by Martinat-Botté et al. (1998). At each examination, the same operator recorded three uterine parameters: the maximum height of the uterus in the ultrasound image or "uterine height" (H), which is the space occupied by the uterus between the abdominal wall and the intestines (Martinat-Botté et al., 1998), the maximum diameter of the uterine horns or "horn diameter” (D) and finally, the percentage of sows with fluid in the lumen of the uterine horns (F).

**Figure 1:**
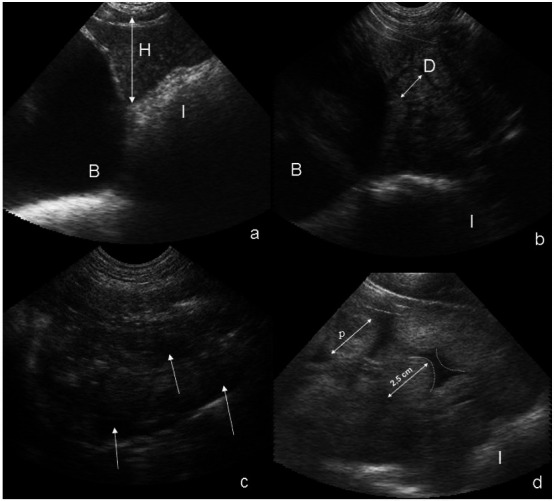
Transabdominal ultrasound images (Falco - Esaote Pie Medical - 5 MHz micro-convex probe) showing bladder (B) intestine (I) and (a) uterine height (H), (b) uterine horns diameter (D), (c) presence of lochial fluid in uterine horns,(→) 5 days postpartum and (d) endometritis with an important horn diameter (↔), presence of echogenic fluid or pus (p) in the lumen and a bigger interspace (delimited by the dotted lines) between uterine horns.

To measure the largest value of uterine height and diameter horns, the functions "freeze" and "cineloop” have systematically been used. The first function allows a freeze frame and the second to revisit past images recorded by the scanner. The probe allowed a 12 cm penetration depth and so allowed measuring heights of maximum 12 cm; for values ≥12, the '12' score was recorded. When the measurement of the diameter of the uterine horns was not technically possible (> 12 cm), no D-value was recorded. The presence of fluid in the uterine horns was assessed using scores 0 (absence of liquid) or 1 (presence of liquid). According to Kauffold et al. (2005), transcutaneous ultrasonography is appropriate to diagnose endometritis, based on a heterogenous echotexture, a higher uterine size and intrauterine fluid (Kauffold et al., 2005). In the present study, endometritis was suspected when a higher horn diameter was associated with the presence of liquid in flaky appearance resulting in the detection of suspended particulates or echogenic areas in the uterine lumen and/or a higher interspace between uterine horns. A note was then recorded. In the same way, if a cystitis (or a urinary tract infection, UTI) - associated to the presence of hyperechogenic material in urine - was suspected, it was also recorded. In agreement with the farmers, they were not informed about the results in order not to interfere with treatment or culling decision. Figure 1 shows an ultrasound scan of uterine height, diameter of the uterine horns and intra-luminal fluid as well as an endometritis case.

### Measuring reproductive performance

In order to test the possible effect of the previous performance of the sow (number of total born piglets or TBP) on H, D and F, the previous numeric production data were recorded for each sow (Table 2).

**Table 2:** Reproductive performance values for 642 sows from 9 farms examined by means of transcutaneous ultrasonography scanner (Falco - Esaote Pie Medical - equipped with 5 MHz micro-convex probe) to describe uterine involution. The farrowing rate (%), the mean interval weaning - first insemination (days; mean ± standard deviation), the mean farrowing interval (days; mean ± SD) and the mean number of total born piglets on next litter or TBP+1 (mean ± SD) were measured for each sow in the next parity.

**Farms**	**Number of sows /farm (n)**	**Scanned sows (n)**	**Farrowing rate (%)**	**Mean interval weaning - first insemination (days)**	**Mean farrowing interval (days)**	**Mean TBP+1**
A	105	10	81.2	4.5 ± 0.5	159.9 ± 36.3	15.4 ± 2.3
B	120	35	84.6	7.0 ± 5.7	153.6 ± 15.5	11.8 ± 2.4
C	120	11	75.0	6.3 ± 2.2	158.9 ± 46.8	14.3 ± 4.0
D	120	43	82.9	5.0 ± 0.8	149.8 ± 21.3	13.2 ± 3.0
E	670	147	77.2	5.5 ± 2.5	149.4 ± 8.8	13.0 ± 2.9
F	200	62	83.3	5.3 ± 2.1	146.3 ± 2.6	11.9 ± 3.4
G	340	196	84.9	4.7 ± 1.4	146.2 ± 8.7	12.5 ± 3.1
H	240	132	83.2	5.3 ± 4.1	149.7 ± 11.1	11.5 ± 3.4
I	70	6	80.0	5.4 ± 0.5	149.8 ± 10.2	12.5 ± 3.5

For each animal, the interval weaning - first insemination (IWI), farrowing (n=303) or not (n=79), and the number of total born piglets at the next cycle (TBP+1) were registered, if praticable. To test the effect of uterine involution on IWI, sows were also classified in 3 groups of IWI: ≤ 5 days (IWIa), 6-10 days (IWIb) and >10 days (IWIc).

### Statistical analyses

To evaluate the effect of the number of DPP (fixed variable) on H, D or the F (dependent variables), we used the following mixed linear model:


$$f\left(yi\right)\;=\;µ\;+\;b*DPPi\;+\;ai\;+\;ei$$ (1)

In this expression, yi is the tested parameter (H, D or F) measured on individual i, µ is an overall mean, DPPi is the number of DPP corresponding to measurement i, b is a regression coefficient of yi on DPPi, ai is the individual i effect and ei is a residual. Expression f(.) refers to a function used to transform the character if necessary (f(yi) = yi for H and D, and f(yi) = ln (yi/(1- yi)) = logit(yi) for F). In this model, DPPi is a fixed effect, and ai is a random effect used to correlate observations made on the same individual.

Since the relation between F and b is not linear (logit), the value of b is more easily interpretated as a Odds Ratio (OR). OR linked to F is the ratio between the probability of liquid when DPP is X + 1 on the same probability if DPP is X. This ratio measures the dependence between F and DPP (this measurement is independent of the value of X). It is calculated as OR = exp(b). A value less than 1 means that the probability of having liquid decreases with increasing DPP.

To test the breed effect (fixed variable), H and D’ analyses are similar to that performed for DPP. Since breed is a classification variable, this effect is tested using a Snedecor F statistics. For the F parameter, the generalized mixed linear model including the individual random effect did not converge. We therefore used the results of the logistic model without the individual effect. In this logistic procedure, we tested the effect of breeds using a chi-square.

We used similar models to test the effect of fixed variables parity and TBP : a mixed linear model with a random effect on the individual was also used to analyse the parameters H and D (dependent variables). For F (dependent variable), a logistic regression tested the effect of parity. We either considered TBP as a classification or a continuous variable. Only the model with a continuous TBP converged for H and F.

The effect of breed was tested using an univariable model and significant differences were shown in some cases. However, given that all breeds were not present in all herds (Table 1) nor in all parity groups, only the analysis of factors parity group and days postpartum in a multivariable model was possible.

To evaluate subsequent sow's performance dependence on H, D and F (fixed variables), we used mixed linear models (generalized for F) with a random effect of the individual sow. In these models, the IWI and the TBP+1 at the next production cycle were treated as continuous (dependent) variables whereas the 3 groups of IWI (a, b, c) and groups of farrowing (yes or no) were considered as classification (dependent) variables.

All these analyses were performed using the Mixed or Logistic procedures of SAS (version 9.3). Results were expressed as mean ± standard errors. Values < 0.05 were considered as statistically significant (two-sided testing).

## Results

### Effect of days postpartum

**Figure 2:**
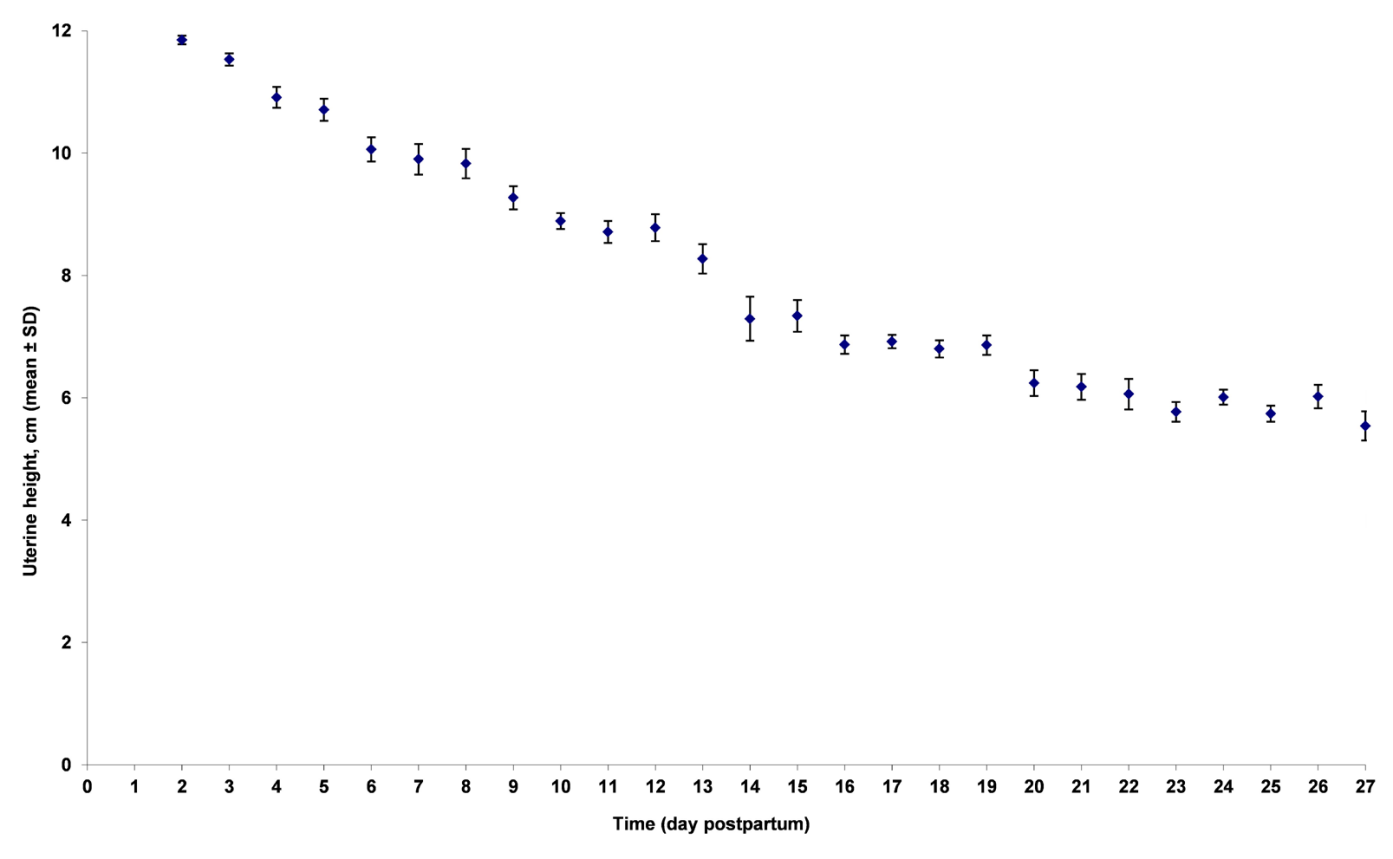
Effect of time on uterine height (in cm; mean ± standard deviation) measured on 642 sows from 9 herds. Sows were ultrasonographically scanned 3 to 4 times at 7 days interval (Falco, Esaote Pie Medical, equipped with a 5 MHz micro-convex transabdominal probe).

**Figure 3:**
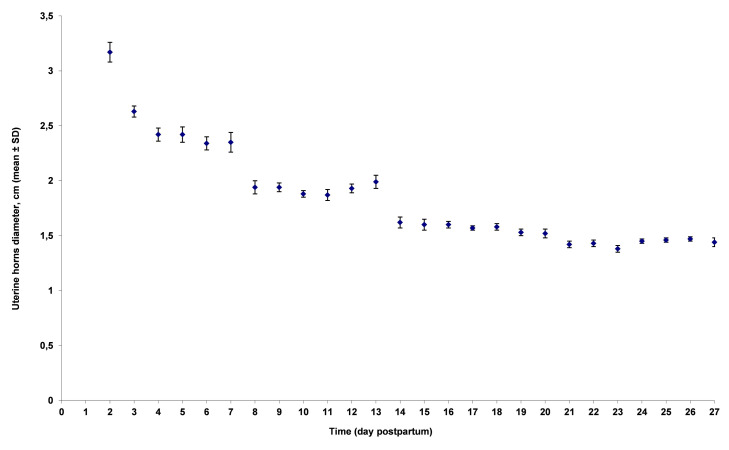
Effect of time on uterine horns diameter (in cm; mean ± standard deviation) measured on 642 sows from 9 herds. Sows were ultrasonographically scanned 3 to 4 times at 7 days interval (Falco, Esaote Pie Medical, equipped with a 5 MHz micro-convex transabdominal probe).

During lactation (3-4 weeks), uterine height (H) decreased 0.25 cm per day (Figure 2), from 11.0 ± 1.6 (mean ± SD) during the first week to 5.9 ± 1.5 cm during the last week (p<0.001) and horn diameter (D) 0.05 cm per day (Figure 3), from 2.6 ± 0.7 to 1.4 ± 0.2 cm (p<0.001), respectively. Between days 1-7, H and D decreased significantly faster, *i.e.* respectively 0.38 cm (95% confidence interval (95% CI): 0.31 to 0.45; p<0.0001) and 0.20 cm (95% CI: 0.16 to 0.24; p<0.0001) per day than between days 22-28, *i.e.* respectively 0.02 cm (95% CI: -0.05 to 0.10; p=0.49) and 0.00 cm (95% CI: -0.01 to 0.02; p=0.75) per day.

**Figure 4:**
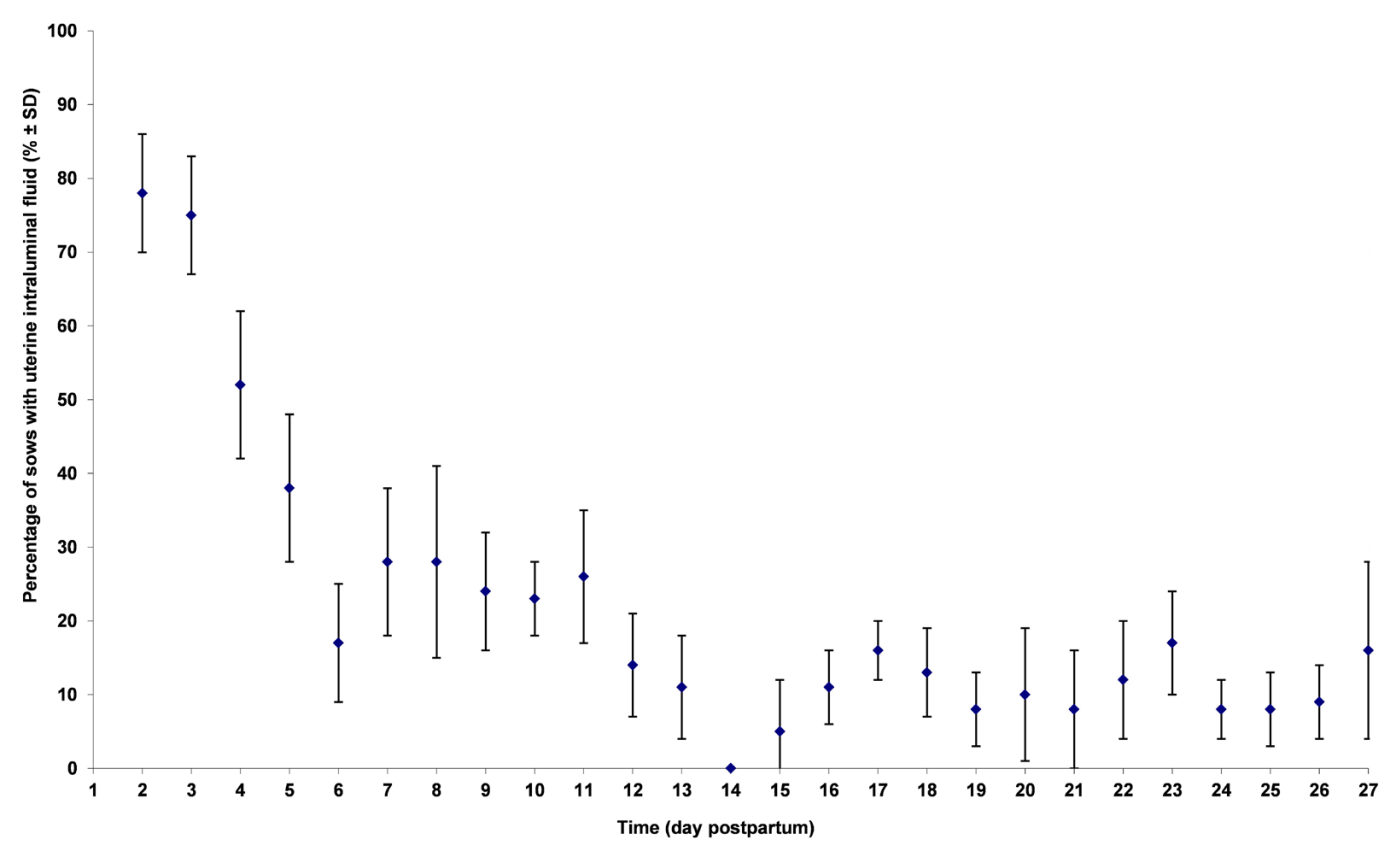
Effect of time on percentage of sows with uterine intraluminal fluid (score >0) measured on 642 sows from 9 herds. Sows were ultrasonographically scanned 3 to 4 times at 7 days interval (Falco, Esaote Pie Medical, equipped with a 5 MHz micro-convex transabdominal probe).

F (Figure 4) decreased significantly (p<0.0001) from 78% at the beginning to 16% at the end of lactation (Odds ratio OR = 0.87, 95% CI = 0.85 to 0.88). Between days 1-7, F decreased significantly (p<0.001) faster (OR = 0.56, 95% CI = 0.50 to 0.64) than during the last week of lactation (OR = 0.93; 95% CI = 0.78 to 1.11; p=0.41).

### Effect of breed

Results show no effect for H and D but for F, there was a significant difference between breeds (p<0.001). In commercial maternal line 1 and 2, the percentage of sows with F was significantly lower than in K+. In CML 4, F was higher than in K+ but the number of CML 4 sows was very low (n=4).

### Effect of parity

The mean parity of the 642 scanned sows was 4.1 ± 2.7. During lactation (3-4 weeks), no significant difference was found between the parity groups for H and D. In regards to F, group C *vs.* A (OR = 1.97; 95% CI = 1.54 to 2.51) and group C *vs.* B (OR = 1.88; 95% CI = 1.48 to 2.39) were significantly different: F was higher in sows of parity ≥6.

**Figure 5:**
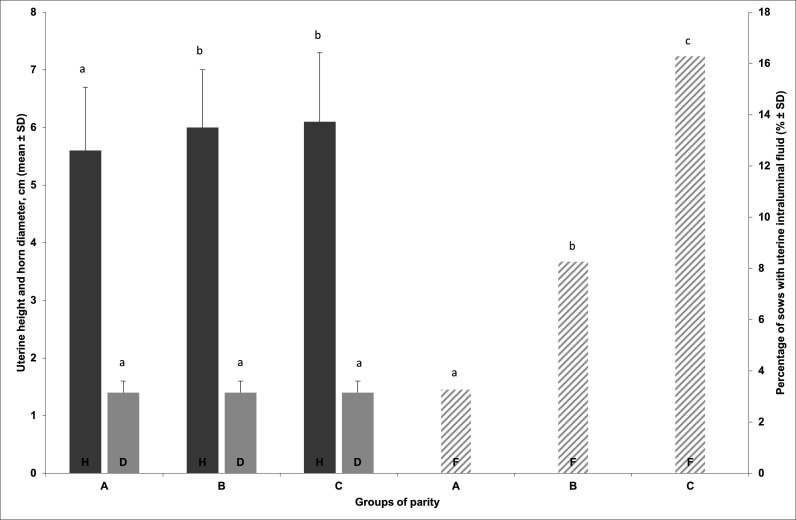
Uterine height (H), uterine horns diameter (D) (in cm; mean ± standard deviation) and percentage of sows with uterine intraluminal fluid (F; score =1) measured during the last week of lactation (days 22-28) on 214 parity 1-2 sows (group A), 242 parity 3-5 sows (group B) and 172 parity ≥ 6 sows (group C). Sows were ultrasonographically scanned (Falco, Esaote Pie Medical, equipped with a 5 MHz micro-convex transabdominal probe). a, b, c: Groups of parity with value of H, D or F without a common superscript letter differ significantly.

During the last week of lactation (days 22-28), H values were 5.6 ±1.1, 6.0 ± 1.0 and 6.1 ± 1.2 cm for the groups of parity A, B and C, respectively. H of parity groups B and C were significantly higher than values observed for group A (p=0.007). There was no significant difference between groups B and C. For D, there was no significant difference between the parity groups. In regards to P, groups B *vs* A (OR = 2.53; 95% CI = 1.04 to 6.14), C *vs* A (OR = 5.74; 95% CI = 2.42 to 13.62) and C *vs* B (OR = 2.42; 95% CI = 1.29 to 4.52) were significantly different: F was significantly higher in sows of higher parity. Figure 5 presents H, D and F values for the parity groups A, B, C measured between days 22-28 postpartum.

### Effect of the litter size

The average TBP of the 642 scanned sows was 11.9 ± 3.2 and they weaned on average 9.6 ± 1.9 piglets. In this study, the litter size did not influence significantly parameters H, D and F.

### Metritis, cystitis/UTI diagnosis and/or fetal retention

During the study, 11 sows showed a cystitis/UTI suspicion associated with the presence of hyperechogenic material in the bladder. Fifteen sows presented in uterine lumen, an abnormal “fluffy” or echogenic fluid accumulation leading to a suspicion of endometritis. Table 3 shows, for these 15 sows, parity, number of days postpartum at the time of observation, IWI, litter size at next farrowing (parity +1) and/or the culling reason given by the farmer, if available.

**Table 3:** Data of 15 sows with suspicion of metritis or diagnosis of foetal retention among 642 sows from 9 farms examined by means of transcutaneous ultrasonography scanner (Type Falco - Esaote Pie Medical - equipped with a micro-convex probe with a frequency of 5 MHz).

**Sows**	**Parity**	**Day of examination** **a**	**Interval weaning – 1st AI (days)**	**Total born piglets** **b**	**Sow culling reasons**
**1**	5	2	4	13	
**2**	4	2	5	11	
**3**	3	3	5	7	
**4**	7	3	4	11	
**5**	7	6	5	7	
**6**	12	7	4	7	
**7**	3	10	21		Not pregnant
**8**	2	10	4	10	
**9**	3	12	5	13	
**10**	1	13	13	4	
**11**	9	11 and 20			Productivityc /oldd
**12**	13	10 and 24			old^d^
**13**	2	24	4		abortion
**14**	2	25	5	7	
**15**	5	18 and 26	5		metritise

^a^Number of days after farrowing. ^b^Litter size (total born piglets) at next farrowing. ^c^Culled sow for low productivity. ^d^Culled sow for old age. ^e^Fetal retention.

Six sows suspected of endometritis during the first week of lactation gave birth to between 7 and 13 TBP+1. Among the eight sows suspected of endometritis during the second or third week of lactation, two were culled due to abortion or not pregnancy, two were culled because of old age and/or low productivity and the four others gave birth to between 4 and 13 TBP+1. One sow presented fetal retention. When performing 3 consecutive transcutaneous ultrasonographies (12, 18 and 26 days postpartum), H was greater than 12 cm and D increased from 2.7 cm (12 days postpartum) to more than 12 cm in the last two examinations. Each time, an important presence of fluid was detected. Fetal retention was diagnosed during the second examination and confirmed at the third. This sow weaned 6 piglets 27 days postpartum. It was inseminated 5 days after weaning and returned in estrus 21 days later. A second insemination was conducted at that time. Finally the sow was culled due to repeat breeding and vulvar discharge 67 days after weaning.

### Relationship between uterine involution and the next performance of sows

Among the 428 individuals retained to study the relationship between uterine involution and subsequent reproductive performance, 313 sows gave birth after one insemination (73.1%), 35 sows were re-inseminated (8.2%), 46 sows were culled after insemination (10.7%) and 34 sows were culled without insemination (8.2%). Amongst 313 sows, the mean litter size was 12.4 ± 3.2 TBP. Among the 35 sows that were re-inseminated, 21 gave birth after a second or a third insemination; 2 sows were re-inseminated after an abortion and one of them had a litter.

Culling of sows that had been inseminated after weaning was decided by the breeder because of the following reasons: no pregnancy (24), lameness (6), abortion (3), disease (1), old age (1) or vulvar discharge (2). Two sows died and six were culled without known cause. The culling of sows that were not inseminated after weaning was decided due to undetected oestrus (7), old age or low productivity (11) or lameness (4). A sow died and eleven sows were culled without known causes.

Among 428 sows weaned 22 to 28 days postpartum, no significant relationship was found between the three uterine parameters measured at the end of lactation and IWI. Similar results were found with classes IWIa, b and c. No significant relationship was found between H and P parameters measured at the end of lactation and the farrowing rate nor with the number of TBP at the next reproductive cycle.

A significantly lower D (- 0.13 cm) was measured in sows that farrowed at the next cycle (p=0.04). A significantly higher D (+ 0.01 cm) was measured in sows with one more TBP+1 (p=0.01).

## Discussion

This study has shown that uterine height decreases almost linearly between day 2 and day 23 postpartum 0.25 cm per day. Maximal measures of 12 cm were observed immediately postpartum. As the probe used in the present study allowed measuring heights of maximum 12 cm, it is possible that uterine height were underestimated at that time. After 23 days, the uterine height was about 6 cm. Similar results, with heights equal to 12, 7 and 6 cm at day 1, 15 and 21 postpartum, respectively, were described by Lee et al. (2000). In the same way, the uterine horns diameter decreased very quickly, reaching a minimum value of 1.4 cm 21 days postpartum. In comparison Meile et al. (2020) have reported uterine horns diameters of 1.0 or 0.9 cm 28-29 days postpartum. Differences observed could be explained by the methodologies: in the present study, maximum horns diameter was measured while Meile et al. (2020) made the average of three measurements. At 21 days postpartum, the percentage of sows still having intra-luminal liquid was 8%. The uterine involution seemed complete after the 21st day postpartum, corresponding to the results of former studies (Slama et al., 1999; Martinat-Botté et al., 1998). Uterine height and horns diameter measured between days 21 and 28 postpartum are complementary to estimate accurately uterine involution: uterine height gives a first and global approach whereas horns diameter provides more detailed and precise information. The detection of (a large amount of) intra-luminal liquid one week postpartum or later is indicative of a delayed involution associated with endometritis (Kauffold et al., 2019) and/or fetal retention.

According to EU legislation, piglets can be weaned 21 days postpartum if special facilities are available for the weaned piglets. At that moment, the uterus seems morphologically ready for a next pregnancy. Svajgr et al. (1974) have shown that the pregnancy rate 28 days after insemination decreases significantly when weaning is performed at 2 or 13 days *versus* 24 or 35 days postpartum. In a study comparing weaning at 10 versus 20 days postpartum (Marsteller et al., 1997), incomplete uterine involution in sows weaned at 10 days was probably the cause for decreased embryo survival and weight of the embryos. Possibly, it also explains the increased IWI. Willis et al. (2003) referred to lack of “adequate uterine environment” when explaining the low rate of embryo survival observed in sows weaned at 14 days postpartum.

The parity of the sow had an influence on uterine height and the presence of fluid in uterine lumen. A higher parity was associated with a higher uterine height after complete involution as well as with a greater probability to find fluid in the uterine lumen anytime postpartum. Consequently, it seems that the volume of the sow uterus increases with parity. As there is no effect of parity on the horns diameter, the higher volume of uterus observed in older sows seems to be associated with a higher length of uterine horns. Meile et al. (2020) measured only one of these three ultrasound parameters - *i.e.* uterine horns diameter and suggested that the decrease of horns length rather than horn diameter is the major contributor to decreased uterine weight/volume during involution. The present study, combining both uterine height and horns diameter measures has shown the major role played by the reduction of the uterus length.

The genetic type (CML 1 and CML 2 versus K +) seemed to have an influence on the presence of liquid in the uterine lumen. However, the significant interaction between breeds and herds on the three studied uterine parameters should be taken into account, meaning that the effect of breed depend on the herd. The distribution of breeds in the different farms did not allow to test the effect of genetic types regardless of herds.

A prostaglandin systemic treatment (Dinolytic^®^) given to all sows 36 h postpartum in farm G might influence the puerperium. We therefore used an additional statistical model including a "prostaglandin treatment" effect in addition to the tested effect (these analyses are not presented in the present paper). The results of these analyses were very similar to those obtained from the models not incorporating the prostaglandin treatment. This is why we did not include it in the final model.

The present study did not show an influence of litter size on the three studied parameters. In the same way, this study did not demonstrate a clear relationship, applicable in the field, between uterine parameters measured during the last week of lactation and the subsequent reproductive performance of sows. Other factors than the uterine involution such as the ovarian activity, the time of insemination, or the quality of semen can influence the pregnancy rate and litter size (Lawlor and Lynch, 2007). Also other factors such as sow farrowing duration lasting more than 300 minutes (Oliviero et al., 2013), have been associated with a lower subsequent fertility. These factors might explain why no significant associations were found in the present study.

Using a convex probe - and even a micro-convex probe - rather than a linear probe to achieve transabdominal ultrasounds allows the operator to put less pressure on the abdomen and to get better skin contact (Maes et al., 2006). As these authors pointed out, the frequency of a probe is inversely related to the depth of penetration and positively correlated to the resolution of the ultrasound image. The choice of a frequency of 5 MHz is justified by the need to obtain an optimal resolution image while allowing reaching sufficient depth - although limited to 12 cm - to observe the whole uterus (excluding the frequency of 7.5 MHz). A 3.5 MHz probe would probably not have allowed to measure precisely the diameter of the uterine horns and to detect easily the presence of fluid in the uterine lumen. The "cineloop" function that allows coming back on the last sequence of stored images was essential for this type of examination. Indeed, the "freeze" function and then the "cineloop" allowed choosing easily the most appropriate images to make measurements.

From a practical point of view, this ultrasound examination of uterine involution in the farrowing room is more laborious and time-consuming than pregnancy diagnosis by ultrasound because of the different housing conditions of the sows in the farrowing house compared to the gestation unit. It also requires more advanced ultrasound devices. The sow must be calm and relaxed at the time of examination, which is not always obvious when someone approaches the offspring. Similarly, if the sow is trying to defecate or urinate, posture and contractions of the abdominal muscles make ultrasound testing impossible. In the present study, examination of each sow lasted approximately 4 to 5 minutes.

In this study, one person performed all the measures, so it was not possible to compare the potential influence of the operator. Having the same person for all measurements had on the other hand the advantage that there was not inter-observer variation that could have biased the results.

The uterine height reflects the importance of the uterine volume. This essentially corresponds to the volume of uterine horns, since the uterine body is relatively short, measuring approximately 5 cm (Pavaux, 1982). This measure may be influenced by two factors: the volume of the abdominal cavity and the viscera it contains. So a urinary bladder filled with urine (which is often the case when the sow stands up for examination) can move laterally the uterine horns. This is especially the case towards the end of uterine involution process. To better estimate the volume occupied by uterus, it would be interesting to measure, perpendicular to the uterine height, the maximum distance occupied by uterus between the bladder wall and the intestines. Another option could be to measure the largest area occupied by the uterus in the ultrasound image, a parameter already used in the past (Martinat-Botté et al., 2003).

Physiologically, normal discharges (lochia) are no more visible 3 to 4 days after farrowing (Slama et al., 1999). Waller et al. (2002) showed that vulvar discharges (> 50 ml per day) after more than 6 days postpartum are associated with decreased pregnancy and farrowing rates. In addition, when these affect primiparous sows, they have also a negative effect on the litter size of the next cycle. The presence of abundant and persistent flow may help to diagnose a severe endometritis. Indeed, the amount of vaginal discharge is indicative of acute endometritis (Grahofer et al., 2021). In this study, a small number of sows were suspected of endometritis (2%). The low number of such problematic sows did not allow to assess relevant associations between ultrasound measures and reproductive disorders. It would be interesting in the future to target some farms with vulvar flow and/or reproductive performance problems in order to link the clinical signs to ultrasound measures of uterine involution. This approach would allow investigating a possible prospective value to uterine involution measurements.

Ultrasound scan revealed a possible cystitis/UTI of 11 sows. Cystitis has been shown to be negatively associated with the subsequent reproductive performance (Martineau and Almond, 2008). Its early detection during the lactation represents a significant advantage. Kauffold et al. (2010) have indeed shown that moderate to high amounts of sediment in the urine of sows seem to be indicative of UTI. Sediment can be visualized by transrectal and transcutaneous scanning. As the first objective of the present study was not to detect or study UTI, no complementary analyses were performed. This warrants further studies.

## Conclusion

The use of transabdominal ultrasonography in lactating sows allows describing the uterine involution under field conditions by measuring uterine height, horns diameter and the presence of liquid in the uterine lumen. The present results demonstrated for the first time the major role of the length of uterine horns to explain the higher volume of uterus in older sows. The scanning also allowed diagnosing a possible uterine or bladder infection or even a feto-placental retention. This examination should be scheduled preferentially during the last week of lactation. This study did not allow to predict the next reproductive performance of scanned sows. Further research is needed to investigate the predictive value of transabdominal scanning of uterine involution for early detection of reproductive performance in farms with a higher prevalence of reproductive problems.
